# The Biological Intersection Between Chemotherapy-Related Cognitive Impairment and Alzheimer Disease

**DOI:** 10.1016/j.ajpath.2024.12.013

**Published:** 2025-01-23

**Authors:** Matthew Torre, Camila A. Zanella, Mel B. Feany

**Affiliations:** ∗ Department of Pathology and Immunology, Baylor College of Medicine, Houston, Texas; † Jan and Dan Duncan Neurological Research Institute, Texas Children’s Hospital, Houston, Texas; ‡ Center for Alzheimer’s and Neurodegenerative Diseases, Baylor College of Medicine, Houston, Texas; § Department of Pathology, Brigham and Women’s Hospital and Harvard Medical School, Boston, Massachusetts

## Abstract

Alzheimer disease (AD) is the most common type of dementia and one of the leading causes of death in elderly patients. The number of patients with AD in the United States is projected to double by 2060. Thus, understanding modifiable risk factors for AD is an urgent public health priority. In parallel with the number of patients with AD, the number of cancer survivors is estimated to increase significantly, and up to 80% of cancer patients treated with chemotherapy will develop cognitive deficits, termed chemotherapy-related cognitive impairment. This review discusses biologically plausible pathways underlying both disorders, with the goal of understanding why a proportion of chemotherapy patients may be at higher risk of developing AD. Highlighted are the *E4* allele of the apolipoprotein E gene, neuroinflammation, oxidative stress, DNA damage, mitochondrial dysfunction, neuronal and synaptic loss, cellular senescence, brain-derived neurotrophic factor signaling, white matter damage, blood–brain barrier/vascular dysfunction, tau pathology, and transposable element reactivation.

Alzheimer disease (AD) is the most common cause of dementia. AD affects an estimated 10.9% of the population in the United States aged ≥65 years, representing 6.9 million people, which is projected to double by 2060.^1^ Because AD is the fifth leading cause of death in this age demographic and is a major source of patient morbidity,^1^ understanding modifiable risk factors for AD is an urgent public health priority. Consequently, there has been much interest in investigating how history of cardiovascular disease, metabolic syndrome, traumatic brain injury, exposure to air pollution, and COVID-19 infection, among other factors, predict future risk of developing AD and related dementias.^2^ The collective endogenous and exogenous environmental risk factors for AD have been termed the “AD exposome.”^3^

Concurrent with the AD exposome, chemotherapy-related cognitive impairment (CRCI) has been a rapidly growing area of research. As of 2024, there are >18 million cancer survivors in the United States,^4^ and this number is expected to increase to 22.1 million by 2030.^5^ Up to 80% of cancer patients treated with chemotherapy have cognitive deficits.^6^ CRCI is functionally significant, resulting in poorer job performance and reduced quality of life and daily functioning.^7^ Although most patients with CRCI show improvement over time, CRCI and its associated radiologic changes, including decreased total brain volume and gray matter volume, can persist for years after cessation of therapy.^8^^,^^9^ The cognitive domains most often affected in CRCI include memory, attention, and executive function,^10^ similar to AD, although the extent of impairment is more modest in CRCI.^11^ In addition to prima facie overlapping symptoms, CRCI and AD share common risk factors, including advanced age, reduced cognitive reserve, and presence of the *E4* allele of the apolipoprotein E (*APOE*) gene.^12^, ^13^, ^14^, ^15^, ^16^

Because of the clinical significance of AD and CRCI, the projected increase in affected patients, and the clinical overlap, it is compelling to consider chemotherapy exposure as a potential risk factor for AD. Given the epidemiologic data, which generally report an inverse correlation between history of cancer/chemotherapy and AD/dementia, it is likely that only a subset of chemotherapy patients may be at higher risk. The goal of the current review was to discuss pathways connecting AD and CRCI (Figure 1 and Table 1) and highlight new research opportunities and potential therapeutic strategies.

**Figure 1:**
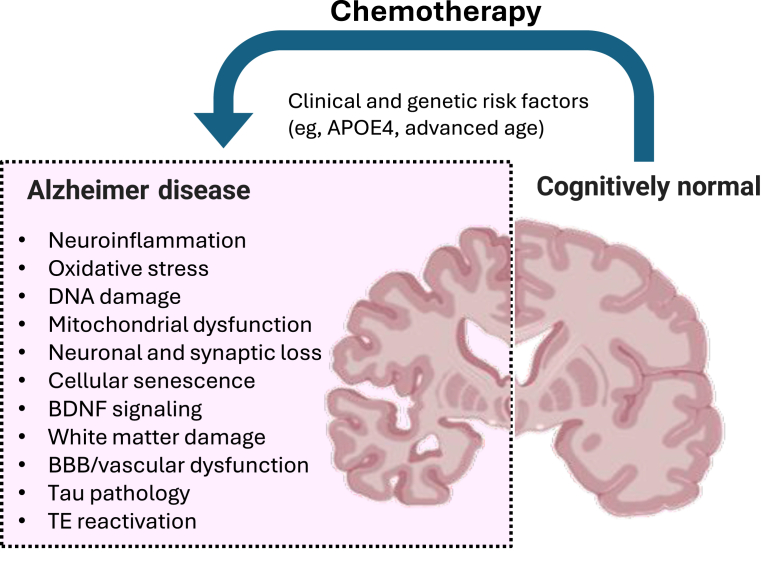
Pathways at the intersection between Alzheimer disease and chemotherapy-related cognitive impairment. Alzheimer disease and chemotherapy-related cognitive impairment have common clinical and genetic risk factors [eg, apolipoprotein E4 (*APOE4*) status, advanced age] and plausibly share biological pathways [eg, neuroinflammation, oxidative stress, DNA damage, mitochondrial dysfunction, neuronal and synaptic loss, cellular senescence, brain-derived neurotrophic factor (BDNF) signaling, white matter damage, blood–brain barrier (BBB)/vascular dysfunction, tau pathology, and transposable element (TE) reactivation]. Thus, chemotherapy exposure may modify the risk of Alzheimer disease and the rate of cognitive decline in Alzheimer disease in at least a subset of patients.

**Table 1:** Intersecting Pathways in AD and CRCI: The Role of Chemotherapeutic Agents

Intersecting pathways in AD and CRCI	Summary of how chemotherapeutic agents potentially contribute to the intersecting pathways in AD and CRCI
Neuroinflammation	• Direct activation of microglia • Elevation of circulating proinflammatory cytokines that may cross the BBB • Altered microbiome • Induction of senescence
Oxidative stress, DNA damage, and mitochondrial dysfunction	• Native mechanisms of action of chemotherapeutic agents • Elevation of circulating proinflammatory cytokines that may cross the BBB • Induction of neuroinflammation and senescence
Neuronal and synaptic loss	• Induction of apoptosis and direct cytotoxic effects in vulnerable cell populations, including precursor/progenitor cells • Reduced neurogenesis • Induction of neuroinflammation, senescence, oxidative stress, and altered adenosine signaling and BDNF signaling
Cellular senescence	• Induction of DNA damage, oxidative stress, mitochondrial dysfunction, and neuroinflammation
BDNF signaling	• Reduced BDNF signaling, possibly secondary to increased inflammation
White matter damage	• Direct cytotoxic effects • Impaired oligodendrogenesis • Induction of neuroinflammation and altered BDNF signaling
BBB integrity and vascular dysfunction	• Elevated systemic inflammation may reduce BBB integrity • Reduced microvascular density and neurovascular coupling • Increase in cardiovascular comorbidities • Induction of senescence in endothelial cells
Tau	• Increase in phosphorylated tau and total tau
Beta amyloid	• Increase in neuroinflammatory response to beta amyloid plaques
Reactivation of transposable elements	• Altered chromatin structure resulting in reactivation of transposable elements

AD, Alzheimer disease; BBB, blood–brain barrier; BDNF, brain-derived neurotrophic factor; CRCI, chemotherapy-related cognitive impairment.

## Common Genetic Risk Factors for AD and CRCI

CRCI and AD share genetic risk factors. The most consistently reported shared genetic risk factor is the *APOE4* allele. The *APOE* gene has three allelic forms (*APOE2*, *APOE3*, and *APOE4*) that encode different structural isoforms of the ApoE lipid-binding protein. *APOE4* is the greatest genetic risk factor for late-onset AD, with homozygous carriers having a 15-fold increased risk, whereas *APOE2* is protective against AD. The pathophysiological role of *APOE4* in AD has been reviewed elsewhere,^17^ but biological processes include beta amyloid (Aβ) clearance, tau phosphorylation, neuroinflammatory response, vascular and blood–brain barrier (BBB) function, and lipid metabolism. *APOE4* is also associated with higher risk of CRCI in cancer survivors,^15^^,^^16^^,^^18^^,^^19^ and one study additionally showed that *APOE2* carriers are resilient against CRCI.^20^ *APOE4* also confers increased risk of CRCI in mouse models. Compared with transgenic *APOE3* knock-in mice, transgenic *APOE4* knock-in mice exhibit worse cognitive function and volumetric losses in brain regions, including the frontal cortex and hippocampus, after chemotherapy.^21^^,^^22^

Emerging data suggest that a single nucleotide polymorphism in the translocase of outer mitochondrial membrane 40 homologue gene (*TOMM40*) may also be a shared genetic risk factor. The *TOMM40* region rs10119 single nucleotide polymorphism has been associated with cognitive impairment in patients with breast cancer treated with chemotherapy^23^ and is a reported risk factor single nucleotide polymorphism in AD.^24^^,^^25^

More generally, single nucleotide polymorphisms in genes involved in DNA damage repair and oxidative stress are associated with different cognitive trajectories in breast cancer patients after adjuvant therapy (aromatase inhibitor and/or chemotherapy).^26^ Genetic risk factors for these same pathways have also been identified in AD.^27^

Limited and conflicting evidence exists for other potential genetic risk factors that may be shared by AD and CRCI. These include polymorphisms in the brain-derived neurotrophic factor (*BDNF*) gene^19^^,^^28^, ^29^, ^30^ and the catechol-o-methyltransferase (*COMT*) gene.^31^, ^32^, ^33^, ^34^

## Biologic Pathways Shared by AD and CRCI

### Neuroinflammation

Neuroinflammation is mediated by multiple cell types, including microglia, astrocytes, and peripheral immune cells, and is characterized by the production of cytokines, chemokines, and reactive oxygen species. The role of neuroinflammation in AD is an active area of research,^35^^,^^36^ but the current review highlights several findings germane to the overlap with CRCI. Neuroinflammation is an early neuropathologic feature of AD and contributes to AD disease progression. Chronic neuroinflammation promotes a deleterious proinflammatory milieu in the central nervous system and an increase in synapse pruning; it may propagate the spread of pathologic tau and Aβ. Circulating proinflammatory cytokines, including tumor necrosis factor-α, are elevated in patients with AD^37^ and are thought to cross the BBB to promote neuroinflammation.

Given the role of neuroinflammation in AD, it is plausible that diseases or conditions that cause neuroinflammation and systemic inflammation will exacerbate AD. In support of this hypothesis are data showing that acute inflammatory episodes with elevated tumor necrosis factor-α (eg, infection, injury) are predictive of cognitive decline in patients with AD^37^ and that some AD rodent models with increased systemic inflammation secondary to lipopolysaccharide administration show increased Aβ and tau pathology.^38^ In addition, neuroinflammation induced by chronic systemic inflammation is thought to be one of the mechanisms underlying several AD risk factors, including obesity, type 2 diabetes mellitus, and air pollution.^38^^,^^39^

Importantly, chemotherapy results in increased neuroinflammation and systemic inflammation. CRCI animal models^40^, ^41^, ^42^, ^43^, ^44^, ^45^, ^46^ have consistently shown increased neuroinflammation and/or proinflammatory cytokines in the brain after exposure to multiple different chemotherapeutic agents, with one model showing activated microglia persisting months after treatment.^42^ Reducing neuroinflammation by clearing the microglia rescued cognition in chemotherapy-treated mice,^42^^,^^43^^,^^45^ highlighting the deleterious effect of neuroinflammation in CRCI. To our knowledge, although human neuropathologic studies quantifying neuroinflammation in patients with CRCI compared with control patients have not been published, reactive gliosis has been described in the context of chemotherapy-induced leukoencephalopathy.^47^

Chemotherapy induced-neuroinflammation is likely caused by multiple mechanisms. Methotrexate, for example, crosses the BBB and directly activates the microglia, resulting in chronic neuroinflammation.^42^ In addition, as previously mentioned, circulating proinflammatory cytokines are thought to incite neuroinflammation. Circulating proinflammatory cytokines such as IL-6 and tumor necrosis factor-α are elevated in chemotherapy patients^48^ and correlate with cognitive impairment^49^^,^^50^ and decrements in hippocampal volume.^49^ Mice treated with chemotherapy also have elevated circulating proinflammatory cytokines, including tumor necrosis factor-α,^51^ which are associated with impaired memory.^52^ Moreover, chemotherapy alters the microbiome,^53^ and the microbiome and microbial metabolites have been shown to modulate neuroinflammation.^54^ Alterations in microbiome composition have also been reported in AD and in preclinical AD.^55^

In summary, neuroinflammation is an important neuropathologic feature of AD and likely plays a role in the progression of AD. Chemotherapy exposure induces neuroinflammation via both indirect mechanisms (eg, peripheral circulating cytokines) and direct mechanisms (eg, crossing the BBB to activate resident microglia). Thus, chemotherapy exposure may exacerbate neuroinflammation in AD and mild cognitive impairment, potentially accelerating both cognitive decline and neurodegenerative pathways downstream of neuroinflammation. However, this will need to be tested in AD model systems. In addition, cancer itself is associated with a proinflammatory state,^56^ and thus additional studies in tumor model systems are needed to determine the potential contribution of cancer to neuroinflammation and cognitive impairment.

### Oxidative Stress, DNA Damage, and Mitochondrial Dysfunction

Oxidative stress, DNA damage, and mitochondrial dysfunction are areas of longstanding interest in AD and have been the subject of multiple recent reviews.^57^, ^58^, ^59^ Briefly, AD patients and AD-relevant model systems show elevated oxidative stress, DNA damage, and evidence of mitochondrial dysfunction in the brain,^57^, ^58^, ^59^ and these processes are tightly linked and occur early in AD. Oxidative stress in AD is the consequence of multiple pathways. Pathologic tau stabilizes the actin cytoskeleton, which disrupts mitochondrial dynamics,^60^^,^^61^ resulting in oxidative stress. Aβ and metal ions form complexes that produce reactive oxygen species.^62^ Neuroinflammation and senescence are proinflammatory states that are elevated in AD and also generate oxidative stress. Oxidative stress induces multiple forms of DNA damage.^63^ Mitochondrial DNA is particularly susceptible to oxidative stress–induced DNA damage, and mitochondrial DNA damage ultimately results in the generation of mitochondrial-derived reactive oxygen species.^64^ Rescuing mitochondrial dynamics^61^ and administering antioxidant N-acetylcysteine amide^65^ improved features of neurodegeneration in preclinical models.

Oxidative stress, DNA damage, and/or mitochondrial dysfunction are also elevated in the brain after chemotherapy exposure, as shown by multiple studies in CRCI model systems.^40^^,^^46^^,^^51^^,^^66^, ^67^, ^68^, ^69^, ^70^, ^71^, ^72^ Chemotherapy patients have increased DNA damage and oxidative stress in frontal lobe cortical neurons compared with control patients,^73^ highlighting the clinical relevance of these pathways in chemotherapy-induced neurotoxicity. Although the authors of this review are not aware of studies examining mitochondrial function/structure in the brains of CRCI patients, limited data from nerve biopsies from patients with chemotherapy-induced peripheral neuropathology suggest that mitochondrial morphology is abnormal.^74^

The mechanisms likely underlying oxidative stress, DNA damage, and mitochondrial dysfunction in the context of chemotherapy exposure partially overlap with those of AD and include neuroinflammation and senescence. In addition, chemotherapeutic agents that cross the BBB can directly elevate oxidative stress and DNA damage in the brain through their native mechanisms of action. Chemotherapeutic agents, especially cisplatin, are known to induce mitochondrial DNA damage, inhibit mitochondrial DNA synthesis, disrupt mitochondrial morphology, and impair mitochondrial respiration.^70^ Chemotherapies with negligible BBB permeability likely incite oxidative stress, DNA damage, and mitochondrial dysfunction in the brain through circulating proinflammatory cytokines.^51^ Antioxidant therapy,^46^^,^^70^ nasal administration of mitochondria,^75^ and inhibiting mitochondrial p53 accumulation^72^ improved cognitive function and other neuropathologic features in CRCI rodent models, highlighting the importance of closely linked oxidative stress, DNA damage, and mitochondrial dysfunction in the pathophysiology of CRCI.

In summary, oxidative stress, DNA damage, and mitochondrial dysfunction are important biological pathways in both AD and CRCI. Additional studies are needed to determine whether chemotherapy exposure synergistically elevates oxidative stress, DNA damage, and mitochondrial dysfunction in AD model systems and if *APOE* status^76^ mediates susceptibility to chemotherapy-induced oxidative stress. Furthermore, because AD neurons seem to have a unique mutation signature thought to be secondary to oxidative stress,^77^ it would be interesting to determine if chemotherapeutic agents induce unique mutational signatures in brain cell populations.

### Neuronal and Synaptic Loss

Neuronal loss and synapse loss are key neuropathologic features of AD and contribute to gross and radiologic brain alterations, including loss of total brain volume, cortical and hippocampal atrophy, and ventriculomegaly. Synapse loss is a strong correlate of cognitive dysfunction in AD.^78^ Loss of neurons also correlates with the extent of cognitive dysfunction in AD^79^ and follows the distribution of tau neurofibrillary tangles.^80^ The number of neurons in patients with AD may be reduced by around 50% in both the superior temporal sulcus^79^ and entorhinal cortex (mild AD)^81^ and by 65% in hippocampal field CA1 (severe AD)^81^ compared with control subjects. Impaired neurogenesis has also been described in AD; it seems to be an early feature of disease^82^ and may contribute to memory decline. Preclinical AD models also show impaired neurogenesis^83^ and dramatic reduction of neurons with disease progression.^84^

Neuronal loss and synapse loss are also widely reported following chemotherapy exposure in preclinical models. Rodents treated with chemotherapy show reduced dendritic branches and/or spines in frontal association cortical neurons^52^ and hippocampal neurons,^70^^,^^85^ as well as reduced markers of synaptic integrity in the hippocampus.^44^^,^^86^ Loss of synapses may be due in part to microglial activation.^87^ There were elevated numbers of brain cells, primarily neurons, with caspase activation in a novel CRCI *Drosophila* model.^68^ Mice treated with chemotherapy show increased positivity following terminal deoxynucleotidyl transferase dUTP nick-end labeling (TUNEL) in cells of the hippocampus and dentate gyrus.^85^ In addition, neurogenesis is reduced in chemotherapy-treated rodents,^41^^,^^69^^,^^88^ with evidence of reduced survival of neural precursor cells^89^ and a dramatic increase in the number of TUNEL-positive progenitor cells in the dentate gyrus of chemotherapy-treated mice.^90^ Neural progenitor cells are especially vulnerable to the direct cytotoxic effects of chemotherapy.^90^ Adenosine signaling and oxidative stress may also contribute to reduced neurogenesis and loss of neurons and synapses after chemotherapy.^70^^,^^91^

To the best of our knowledge, formal quantification of synapse and neuronal loss after chemotherapy treatment has not been performed in human brain tissue. However, several radiologic alterations have been described in chemotherapy patients, including volumetric loss of cortical gray matter and hippocampus,^92^ that are compatible with neuronal death and synapse loss.

In summary, neuronal loss and synapse loss strongly correlate with cognitive dysfunction in AD. CRCI model systems also exhibit neuronal loss (including reduced neurogenesis) and synapse loss, with complementary supporting radiologic studies coming from chemotherapy patients. Neuropathologic studies to formally quantify neuronal and synapse loss in chemotherapy patients would be valuable, especially if the data could be correlated with cognitive function. Chemotherapy needs to be administered to AD organismal models to determine if chemotherapy exacerbates neuronal loss and synapse loss and to address if the neuron subtypes most susceptible to AD-related neurodegeneration are also more vulnerable to chemotherapy-induced neurotoxicity.

### Cellular Senescence

Senescence is a heterogeneous form of biological aging characterized by a range of cellular responses, including cell cycle arrest, structural chromatin changes, morphologic alterations, metabolic changes, and expression of a secretome (the senescence-associated secretory phenotype) comprised of proinflammatory cytokines, chemokines, proteases, and growth factors. DNA damage, oxidative stress, telomere shortening, oncogene activation, and mitochondrial dysfunction can all induce senescence. Accumulation of senescent cells results in impaired tissue function, which in the brain can manifest as cognitive deficits.^93^

Senescent astrocytes,^94^^,^^95^ microglia,^95^^,^^96^ neurons (including neurofibrillary tau tangle-containing neurons),^97^, ^98^, ^99^ oligodendrocyte precursor cells (OPCs),^100^ and endothelial cells and pericytes^101^ have been described in the context of AD and related tauopathies in human tissue and/or animal models. Collectively, these diverse senescent cell populations are implicated in numerous pathologic features of AD and AD-related dementias, including neuroinflammation/gliosis,^94^^,^^95^^,^^98^^,^^100^ neuronal loss,^95^^,^^97^ pathologic tau,^95^^,^^97^ Aβ,^100^ mitochondrial dysfunction,^97^ impaired BBB integrity,^101^ and aberrant cerebral blood flow.^97^ Reducing senescent cells improves cognition in AD-relevant animal models.^94^^,^^95^^,^^100^ The SToMP-AD (Senolytic Therapy to Modulate the Progression of Alzheimer’s Disease) clinical trial is currently assessing the safety and efficacy of senescent cell clearing pharmacologic compounds in modulating the progression of AD, highlighting the enthusiasm of exploring senescence as an integral and targetable pathway in AD.^102^

Cellular senescence is also highly relevant to CRCI. Chemotherapy is a known inducer of senescence, and chemotherapy patients have elevated circulating biomarkers of senescence.^103^ Senescent neurons,^104^ microglia,^104^ OPCs,^104^ and endothelial cells^104^^,^^105^ have been observed in CRCI animal models. In one study, investigators used genetic and pharmacologic approaches to ablate senescent cells and showed that these interventions improved cerebral vascular function, BBB integrity, neuroinflammation, and learning and memory.^105^ In another study, transgenic *APOE4* knock-in mice were more susceptible to chemotherapy-induced senescence in the brain compared with *APOE3* knock-in mice,^104^ suggesting that brain cell senescence is mediated by *APOE4* status.

Thus, given the emerging evidence of senescent brain cells in AD and CRCI, the contribution of cellular senescence to the neuropathology and cognitive deficits of these disorders, and the known ability of senescent cells to induce senescence in neighboring cells via senescence-associated secretory phenotype components,^106^ it seems plausible that chemotherapy exposure increases the overall burden of senescent cells in patients with AD or preclinical AD to accelerate cognitive decline and neurodegeneration. Additional studies are also needed to determine if chemotherapy exposure results in a synergistic increase in the number of senescent brain cells in AD model systems and if ablating these senescent cells rescues cognition and improves neurodegeneration. Furthermore, because senescence can be induced by different pathways, and there is significant heterogeneity in senescent cells,^107^ additional studies are also needed to compare the molecular and genetic phenotypes of senescent cells in the contexts of AD and chemotherapy exposure.

### BDNF Signaling

BDNF signaling is involved in many processes relevant to learning and memory, including synaptic transmission, structural/functional plasticity, and survival of mature neurons and precursor cells. Although the clinical significance of the *BDNF* Val66Met polymorphism in CRCI and AD is unclear, the literature consistently reports an association between reduced levels of BDNF with CRCI and AD, as well as a protective effect of increasing BDNF in ameliorating these disorders in preclinical models. BDNF mRNA levels are reduced in the hippocampus of patients with AD,^108^ and the precursor form of BDNF is reduced up to 40% in the parietal cortex of patients with advanced AD.^109^ The reduction in mature BDNF and the precursor form of BDNF seems to be an early event in AD pathogenesis.^110^ Overexpression and hyperphosphorylation of tau, overexpression of Aβ, and systemic inflammation are sufficient to decrease BDNF.^111^^,^^112^ Importantly, BDNF administration improves cognition and other features of neurodegeneration (related to neuronal and synapse loss and neurogenesis) in animal models of AD and aging.^113^^,^^114^ BDNF protects OPCs from Aβ-induced toxicity *in vitro*.^115^

BDNF is also reduced after chemotherapy exposure. In CRCI rodent models, reduced BDNF has been associated with decreased neurogenesis.^69^ Treatment with riluzole, a glutamate modulator that also up-regulates BDNF, improves cognition, neurogenesis, and neuroinflammation in mice given chemotherapy.^116^ Another study found that methotrexate-treated mice have reduced cortical BDNF and that BDNF signaling to OPCs is critical for adaptive myelination, with administration of LM22A-4, a small-molecule partial agonist of the BDNF receptor [tropomyosin receptor kinase B (TrkB)], increasing BDNF signaling and improving cognition and myelination.^117^ In chemotherapy patients, higher plasma BDNF levels at the end of treatment are associated with a lower risk of persistent CRCI.^118^ Peripheral levels of BDNF are also inversely correlated with peripheral levels of multiple proinflammatory cytokines after chemotherapy.^119^ Several studies have found a positive association between peripheral BDNF level and cognition in cancer patients more generally.^120^

In summary, BDNF signaling is reduced in both AD and CRCI, and this reduction likely contributes to loss of neurons and synapses and impairs myelination. Thus, it is plausible that chemotherapy exposure exacerbates BDNF loss in the context of AD to accelerate neurodegeneration, although this needs to be evaluated in future studies. It would also be promising to extend the use of the TrkB partial agonist LM22A-4 to clinical trials with chemotherapy patients. [As an aside, understanding if there are cognitive effects of TRK inhibitors is deserving of long-term study. TRK inhibitors, which target TrkB among other Trks, are being used to treat cancers with neurotrophic Trk (*NTRK*) fusions.] Additional studies are needed to characterize the contribution of BDNF signaling^121^ in mediating the neuroprotective effect of gamma entrainment using sensory stimuli (GENUS)^122^ and to determine if adenosine signaling can be selectively modulated to increase cognitively beneficial BDNF expression.^123^

### White Matter Damage

Multiple white matter abnormalities are observed in AD, with recent data highlighting an important role of myelin dysfunction in the early pathogenesis of AD. White matter damage is associated with reduced memory, executive function, and speed processing.^124^ Radiologically, patients with AD exhibit reduced white matter integrity^125^ and white matter hyperintensities (WMH).^126^ The burden of WMH is predictive of more rapid cognitive decline in patients with mild cognitive impairment.^127^ Histologically, the regions of WMH primarily exhibit gliosis and loss of myelin and axons, indicating that AD-associated WMH are not completely identical to age-related WMH secondary to vascular pathology.^128^ Altered oligodendrogenesis, including OPC numbers, and myelin loss in multiple brain regions have also been described in AD^129^^,^^130^ and may contribute to neuroinflammation.^115^ Preclinical AD models show the importance of BDNF signaling in OPC survival, proliferation, and differentiation^115^ and highlight that pharmacologically up-regulating myelination improves cognition.^129^ Importantly, an emerging body of literature implicates myelin dysfunction/structural deficits as a driver of Aβ deposition in AD,^131^ raising the compelling possibility that demyelinating disorders can potentiate Aβ pathology. Supportive data have been collected in both acute and chronic paradigms of demyelination performed in 5xFAD mice, in which amyloid aggregates were present adjacent to areas of the central nervous system with the most severe demyelination.^131^

Chemotherapy can also result in demyelination and other forms of white matter damage. In severe cases, patients experience fatal chemotherapy–induced toxic leukoencephalopathy, characterized by marked demyelination.^47^ White matter changes, broadly speaking, are frequently observed after chemotherapy,^124^ can persist for years following cessation of treatment in a subset of patients,^132^ and may correlate with the extent of cognitive dysfunction.^133^ Chemotherapy exposure is associated with dysregulated oligodendrogenesis, including OPC levels, in both chemotherapy patients and CRCI mice models.^42^ Loss of myelin is observed following administration of multiple chemotherapeutic agents in mice.^42^^,^^122^^,^^134^ OPCs and oligodendrocytes are vulnerable to chemotherapy-induced toxicity, and sublethal concentrations of chemotherapy can disrupt oligodendrogenesis.^90^^,^^135^ Reducing neuroinflammation restores oligodendrogenesis, myelination, and cognition after chemotherapy,^42^ and increasing BDNF signaling by pharmacologically stimulating the TrkB receptor, which is expressed by OPCs, improves myelination and cognition in chemotherapy-treated mice.^117^

In summary, white matter damage, myelin loss/dysfunction, and altered oligodendrogenesis are recognized alterations in AD and CRCI, with emerging data supporting a key role of these pathways in the pathophysiology and cognitive decline of AD and CRCI. Future studies are needed to determine if myelin-damaging chemotherapeutic agents increase amyloid deposition in AD models and if up-regulating BDNF signaling is neuroprotective in this context. Drugs such as methotrexate and 1,3-bis(2-chloroethyl)-1-nitrosourea that are associated with chemotherapy-induced toxic leukoencephalopathy would be promising chemotherapeutics to test initially. It would also be important to understand if chemotherapy exposure synergistically impairs oligodendrogenesis in AD model systems.

### BBB Integrity and Vascular Dysfunction

Changes to the BBB and to cerebral vascular function in AD are complex and have been reviewed in depth elsewhere.^136^^,^^137^ Vascular neuropathology is seen in the majority of patients with AD^138^ and may contribute to reduced clearance of Aβ in the brain. There is also evidence of increased cerebral BBB permeability, which occurs early in AD pathogenesis^139^ and is more severe in *APOE4* carriers.^140^ Deposition of blood proteins such as fibrinogen into the brain parenchyma contributes to microglial activation and synapse loss.^141^ Another vascular alteration observed early in AD is reduced activity of the P-glycoprotein 1 protein expressed by endothelial cells.^142^ P-glycoprotein 1 removes select toxins and drugs (including the chemotherapeutic agent vinblastine^143^) from the brain and transports them back into blood circulation, thereby limiting the concentration of these substances in the brain. Furthermore, as previously mentioned, senescent endothelial cells have been described in an AD mouse model.^101^

Chemotherapy alters the function of the cerebral vasculature and BBB. It has long been speculated that chemotherapy reduces BBB integrity. As previously described, circulating proinflammatory cytokines are elevated after chemotherapy and can remain elevated long after cessation of therapy; several studies have reported increased BBB permeability in the context of systemic inflammation,^144^ suggesting a mechanism by which chemotherapy may increase BBB permeability. In addition, in a mouse CRCI model, administration of paclitaxel induced senescence in cerebral endothelial cells, contributing to reduced microvascular density, neurovascular coupling (ie, ability to increase cerebral blood flow to metabolically active brain regions), and BBB integrity, with increased leakage of fluorescent tracers up to 500 kDa in molecular weight.^105^ More generally, chemotherapy is associated with multiple cardiovascular complications,^145^ including hypertension and stroke, which are known risk factors for AD and mixed dementia.

The vascular changes observed in AD and CRCI plausibly exacerbate the pathophysiology of the other disorder in a bidirectional way. Additional studies are needed to determine if reduced cerebral perfusion following chemotherapy impairs clearance of Aβ and if activity of P-glycoprotein 1 in AD is sufficiently reduced to cause neurologically consequential higher concentrations of chemotherapeutic agents in the brain.

### Tau

The putative physiological role of tau involves microtubule stability, cytoskeleton organization, and intracellular trafficking. The ability of tau to bind to microtubules is regulated by posttranslational modifications, including phosphorylation. Aberrant hyperphosphorylation promotes the dissociation of tau from microtubules and the formation of tau aggregates. These tau aggregates develop into neurofibrillary tangles that are a pathologic hallmark of AD. In AD, the anatomic distribution of neurofibrillary tangles progresses in a stereotyped pattern^146^ that is strongly correlated with cognitive function.^147^ Total tau and phosphorylated tau in the cerebrospinal fluid are useful biomarkers for AD, as well as mild cognitive impairment due to AD.^148^

A growing body of literature also highlights tau in the pathophysiology of CRCI. Chemotherapy patients have increased cerebrospinal fluid total tau throughout treatment.^149^ Furthermore, levels of cerebrospinal fluid total tau or phosphorylated tau measured during administration of methotrexate-based regimens are negatively correlated with cognitive function,^150^^,^^151^ which can be impaired long term after therapy.^150^ A cross-sectional, proof-of-concept pilot study performed tau positron emission tomography imaging with flortaucipir (^18^F) on patients with dementia or mild cognitive impairment after cancer treatment and showed that chemotherapy patients with episodic memory impairment had paired helical filament tau deposition in an AD-like pattern.^152^ Additional support for a role of tau in CRCI comes from mouse models. Endogenous tau clusters^86^^,^^153^ and increased phosphorylated tau staining^71^ have been reported in the hippocampus and entorhinal cortex of cisplatin-treated B6 mice with cognitive deficits; in these studies, therapeutic interventions, including administration of mesenchymal stem cells^153^ and a histone deacetylase 6 inhibitor,^71^ prevented tau pathology and rescued cognitive deficits.

In summary, there is compelling evidence linking chemotherapy exposure to tau pathology in both patients and CRCI mouse models. Additional studies are needed to: i) determine if chemotherapy exposure exacerbates tau pathology and neurodegeneration in tauopathy and AD model systems, ii) mechanistically unravel how chemotherapy-induced tau pathology contributes to CRCI and neurodegeneration, iii) compare the protein structure and posttranslational modifications of chemotherapy-induced tau species with that of AD, and iv) confirm whether chemotherapy patients have increased pathologic tau, which can then be targeted by tau immunotherapies. Notably, different chemotherapeutic agents may have opposite effects on tau. In contrast to the chemotherapeutics previously mentioned such as methotrexate and cisplatin, there is a class of chemotherapies (taxanes, including paclitaxel, which are microtubule-stabilizing agents) that may be protective against pathologic tau.^154^

### Aβ Plaques

Aβ plaques are another pathologic hallmark of AD. However, there are no strong published data supporting a direct role of Aβ in the pathophysiology of CRCI. Instead, limited data support an indirect connection between chemotherapy treatment and Aβ-mediated neurodegeneration. In an amyloid/APOE transgenic mouse model, treatment with doxorubicin resulted in an increase in the astrocytic response to dense core and compact Aβ plaques but not the quantity of Aβ plaques or the levels of Aβ40 and Aβ42.^155^ Thus, there is preliminary evidence that chemotherapy (specifically doxorubicin) does not modulate Aβ pathology itself but rather the neuroinflammatory response associated with Aβ plaques.

### Reactivation of Transposable Elements

Transposable elements (TEs) are DNA sequences that can move throughout a host genome. However, they are typically enriched in regions of constitutive heterochromatin. In AD, there is accumulating evidence that pathologic tau, oxidative stress, and DNA damage contribute to loss of heterochromatin that importantly results in reactivation of TEs causing aberrant cell cycle activation in neurons and, ultimately, apoptosis.^156^^,^^157^ In addition to contributing to neuron death in AD, expression of TEs and toxic intermediates, such as retrovirus-like retrotransposon-derived double-stranded RNA, promotes neuroinflammation in AD.^158^

The role of TEs in CRCI is understudied but is an innovative new direction. Supporting this novel approach in CRCI is a study showing alterations to chromatin structure and increased expression of TEs in hematopoietic stem cells after chemotherapy exposure.^159^ In addition, *Drosophila* treated with cyclophosphamide show complex changes in TE expression that varies by specific TE and time interval from treatment.^160^ Further studies are needed to determine if TE reactivation occurs in the human brain after chemotherapy treatment and if this pathway contributes to CRCI. If so, then it is plausible that chemotherapy exposure exacerbates cognitive decline and neurodegeneration in the setting of AD by increasing the total expression of TEs and subsequent neuron death.

### Epidemiologic Correlation Between AD and CRCI

Given that AD and CRCI share common biological mechanisms and risk factors, one would presume that there are strong epidemiologic data showing an increased risk of AD in chemotherapy patients. However, epidemiologic studies investigating the association between AD/dementia and history of chemotherapy (or history of cancer more generally) typically report an inverse correlation,^161^, ^162^, ^163^, ^164^, ^165^ although the data are mixed.^166^^,^^167^ Biological explanations for the inverse relationship include framing neurodegeneration and cancer at opposite ends of a spectrum (neurodegeneration being characterized by cell death and cancer being characterized by cell survival and proliferation), referencing genes such as *PIN1* and *P53* that are differentially activated in the contexts of neurodegeneration and cancer.^168^ However, survival bias^161^ and delays in dementia diagnosis in cancer patients^169^ may partially contribute to the inverse association.

Importantly, there may be only a subset of chemotherapy patients who are at higher risk of developing AD, highlighting the need to understand differences in cognitive trajectories after treatment. The increased risk of developing AD after chemotherapy could be conditional on patient genotype (eg, *APOE4* allele) and a number of factors such as chemotherapy regimen, cumulative dose of chemotherapy, and age at time of treatment, among others. Some chemotherapeutic agents may protect against AD neuropathology.^154^^,^^170^^,^^171^ In addition, in some patients, the level of cellular stress induced by chemotherapy exposure could conceivably result in a neuroprotective hormetic effect, although this point is purely speculative.

## Conclusions

The number of patients with AD and the number of cancer survivors will increase dramatically over the next decades. Understanding how chemotherapy exposure modulates subsequent risk of AD and the rate of cognitive decline in patients with AD is therefore an urgent public health priority. There are multiple pathways connecting AD with CRCI, including neuroinflammation, oxidative stress, DNA damage, mitochondrial dysfunction, neuronal and synaptic loss, cellular senescence, BDNF signaling, white matter damage, BBB/vascular dysfunction, tau pathology, and TE reactivation, in addition to common genetic risk factors such as *APOE4* status. The authors are particularly interested in understanding how chemotherapy exposure modulates tau pathology and downstream pathways in preclinical models.

Existent epidemiologic data on the association between AD and chemotherapy history highlight the need to understand genotype–environment interactions that underlie cognitive trajectories after chemotherapy exposure. There could still be a subset of chemotherapy patients at increased risk of developing AD, as predicted by the biological overlap between CRCI and AD. Susceptibility to developing AD after chemotherapy exposure could relate to genotype (ie, *APOE* status), specific chemotherapeutic regimen, accumulated dose of chemotherapy, age at time of treatment, and other factors modulating the pathways as described in this review.

To mechanistically investigate genotype–environment interactions, high-throughput, experimentally facile model systems are needed. The aforementioned mechanistic approach should be complemented by prospective longitudinal patient studies with genome sequencing, multi-omic analysis, and interval cognitive, biomarker, and radiologic data to identify clinical and genetic risk factors for AD and AD progression in chemotherapy patients. Ideally, patients in such studies would consent to brain donation so that the clinical and multi-omic data can be correlated with neuropathologic findings. Once there is a better understanding of the shared biological pathways underlying CRCI and AD, the ultimate goal is to enable clinicians to identify chemotherapy patients who are at increased risk of future cognitive decline and prophylactically treat these patients with disease-modifying therapies.

Finally, because many of the pathways highlighted in this review are shared with other neurodegenerative diseases, further studies are needed to investigate the pathophysiological and epidemiologic relationships between chemotherapy exposure and frequency/severity of neurodegenerative diseases other than AD.

## Disclosure Statement

None declared.
